# Facebook Usage and Life Satisfaction

**DOI:** 10.3389/fpsyg.2019.02711

**Published:** 2019-11-29

**Authors:** Stefan Stieger

**Affiliations:** ^1^Department of Psychology and Psychodynamics, Karl Landsteiner University of Health Sciences, Krems an der Donau, Austria; ^2^Faculty of Psychology, University of Vienna, Vienna, Austria

**Keywords:** Facebook, life satisfaction, social networking sites, replication, friends, self-esteem

## Abstract

Social networking sites (SNS), such as Facebook, attract millions of users worldwide by offering highly interactive social communications. Although this has many advantages, previous research has suggested there are also drawbacks (e.g., stalking, addiction, invasion of privacy). The question, therefore, arises as to whether Facebook – currently the largest social network – makes us more satisfied with our lives. In two independent samples (Study 1: *N* = 2,272; Study 2: *N* = 1,459), Facebook users were neither more satisfied with their lives compared to non-Facebook users, nor were Facebook users with more online friends more satisfied compared to those with fewer online friends. On the contrary, Facebook usage duration was negatively associated with life satisfaction, even after controlling for age and sex differences (Study 1: *r* = −0.11, Study 2: *r* = −0.18). Although in general positive effects would have been expected because Facebook should enhance possibilities for social communication and connection, either null-finding or negative associations were found. Results are discussed in light of possible addiction-supporting features of Facebook, which are reflected in Facebook usage patterns (e.g., higher mean duration of Facebook usage per week, larger number of logins to Facebook).

## Introduction

In its early years, the Internet was generally characterized by non-synchronous and unidirectional communication (e.g., reading web pages or sending emails). Today, by contrast, online communication is highly multidirectional and synchronous. We can communicate in real-time with multiple people across the globe at once. This so-called Web 2.0 era is strongly associated with new Internet services enabling this new form of online communication – particularly social networking sites (SNS) (Raacke and Bonds-Raacke, 2008; Nadkarni and Hofmann, 2012). By far the most popular SNS worldwide is Facebook, now having more than 1.7 billion active users (Statistica, 2016). One possible reason for Facebook’s success is based on the human drive to form social bonds and to communicate (Wilson et al., 2012).

Although SNS do have their advantages, studies in many areas of human social cohabitation have described problems associated with SNS use. For example, SNS appear to facilitate jealousy and suspicion in romantic relationships because of the ease of communicating with potential partners (Muise et al., 2009), which can lead to new forms of stalking and harassment (Dreßing et al., 2014). In addition, the workplace is affected when personnel managers use SNS entries to evaluate job candidates (Bohnert and Ross, 2010). Meanwhile, a new sub-form of non-optimal Internet use has emerged – problematic Facebook use (Kuss and Griffiths, 2011; Dantlgraber et al., 2016), eliciting diverse effects on mental health (Frost and Rickwood, 2017). These negative aspects have even led to a countermovement of users purposefully deleting their SNS accounts (called “virtual identity suicide”) (Stieger et al., 2013). Nevertheless, irrespective of SNS’ downsides and advantages in specific domains, the question remains if, for example, using Facebook really makes users more satisfied with their lives compared to non-users (Valenzuela et al., 2009; Oh et al., 2014; Brailovskaia and Margraf, 2016; Tromholt, 2016). This is not only of scientific interest, but also an issue of societal importance that may help shape both public policy (e.g., regulation of SNS) and user decision-making (e.g., whether to remain on SNS).

Life satisfaction has been frequently studied in relation to Facebook usage in general (Błachnio et al., 2016; Kross et al., 2016) and the number of Facebook friends in particular (Tromholt, 2016; Huang, 2017; Vanman et al., 2018). For example, Huang (2017) included eight studies in one meta-analysis and found a small non-significant overall association of *r* = **−**0.03 between life satisfaction and Facebook usage. Evidence from experiments have found a positive effect of non-Facebook usage (*d* = 0.28), i.e., users who quit Facebook for a week had higher life satisfaction than those who kept using Facebook (Tromholt, 2016). However, another experiment found detrimental effects, with non-Facebook users showing lower life satisfaction (*d* = 0.54) after 5 days compared to Facebook users, despite also having lower levels of the stress hormone cortisol (*d* = 0.41) (Vanman et al., 2018).

Nonetheless, scientific evidence remains inconclusive as to whether Facebook usage enriches users’ lives to a point that it renders greater life satisfaction (Valenzuela et al., 2009; Kim and Lee, 2011; Manago et al., 2012; Lönnqvist and große Deters, 2016). Reasons for this inconsistency lay in (1) underpowered studies (i.e., inadequate sample sizes to reach sufficient power for the results to have a chance of being replicated), (2) variation in sample types (i.e., student samples are often used), (3) different designs (mean differences between non-SNS vs. SNS users; correlational designs with SNS users alone), and different conceptual definitions of constructs (e.g., definitions of life satisfaction). In view of these issues, the present studies were aimed at analyzing life satisfaction of Facebook users and non-users in more detail by taking a closer look at possible associations with the number of Facebook friends and the frequency of usage.

The following research questions (RQs) were addressed:

RQ 1: Are Facebook users more satisfied with their lives than non-Facebook users?

RQ 2: Are Facebook users with more Facebook friends more satisfied with their lives than Facebook users with fewer Facebook friends?

RQ 3: Are frequent Facebook users more satisfied with their lives than infrequent Facebook users?

## Methods

### Participants and Procedure

The following design was applied: An initial, large sample (Study 1: *N* = 2,272; for power considerations, see Supplementary Material) was recruited by several research assistants utilizing a convenience sampling approach (i.e., recruiting participants by word-of-mouth through relatives, friends, and friends-of-friends; for results concerning possible non-independence effects, see Supplementary Material). The study was designed to examine whether and how Facebook usage is associated with satisfaction with life, and was part of a larger project. The sample was age-stratified, employing seven age strata (18–20, 21–25, 26–30, 31–40, 41–50, 51–60, 61+) in order to sort an almost equal number of participants into each stratum (the first three strata were narrower than the remaining ones in order to have a more balanced age distribution in each strata). For life satisfaction, the conceptual definition introduced by Diener et al. (1985) was used. Furthermore, the focus was not only on Facebook users, but also on non-Facebook users who were used as a reference group (for results related to measurement equivalence of life satisfaction across both groups, see Supplementary Material). Three Facebook characteristics were assessed: Whether the participant has a Facebook account, and if so, how many online friends they have and the weekly mean Facebook usage for private purposes in hours.

Additionally, an independent sample was recruited (Study 2: *N* = 1,459) based on several independent projects in which questions about Facebook usage and life satisfaction were included (project #1: *N* = 200; project #2: *N* = 80 with retest after 1 week; project #3: *N* = 160; project #4: *N* = 1,019; again, all samples utilized a convenience sampling approach as in Study 1). This study was designed to examine whether the findings from Study 1 could be replicated, a best-practice recommendation in order to shield one’s results against false-positive scientific findings (Open Science Collaboration, 2015). Furthermore, it allows for an examination of the robustness of an effect, presupposing that the replication is successful (Asendorpf et al., 2013). The recruitment protocol was the same as in Study 1, except for project #4, which used an online survey and several online-specific recruitment strategies (invitations to participate sent to mailing lists and social networks, as well as a passive recruitment strategy by posting the link on several research websites).

The aim was to recruit community-based samples and thus a more heterogeneous demographic compared to student samples. Furthermore, because certain groups can be hard-to-reach via online questionnaires (e.g., older people), paper-and-pencil questionnaires were preferred over online surveys.

To obtain a more complete picture of the RQs under investigation, in a subsample of Study 2 (project #4), additional questions about Facebook usage (Facebook login frequency; minutes per session; duration of usage of Facebook) and friends (number of close friends) were asked. Furthermore, to see if the relationships with life satisfaction might generalize to other concepts, measures of self-esteem and loneliness were included.

After data cleaning and application of eligibility criteria (i.e., German-speaking participants; in project #4, 194 English-speaking participants were also recruited; this group was too small for separate analyses and was therefore discarded), 2,121 participants remained in Study 1 and 1,232 in Study 2. All data collection took place using paper-and-pencil questionnaires, except for project #4 (online survey; for descriptive statistics, see Table 1).

**TABLE 1:** Sample descriptives.

	**Study 1**	**Study 2**
*N*	2,121	1,232
Women, *n* (%)	1,189 (56.1%)	723 (58.7%)
Age, range (years)	18–89	18–88
Interquartile range	26–54	24–44
Mean (*SD*)	41.4 (17.0)	34.5 (14.5)
Facebook account, *n* (%)	987 (46.5%)	848 (68.8%)
Number of Facebook friends, range	0–1,400	0–5,000
Mean (*SD*)	196.5 (173.6)	246.7 (263.3)
Median (Interquartilrange)	150 (70–280)	200 (103–328)
Hours on Facebook on average per week for private purposes, range	0–64	0–96
Mean (*SD*)	5.0 (7.0)	5.4 (9.1)
Median (Interquartilrange)	2 (1–7)	2 (1–6)

### Materials

#### Satisfaction With Life Scale (SWLS)

The SWLS (Diener et al., 1985; German translation Glaesmer et al., 2011) is a 5-item scale measuring general satisfaction with one’s life (Cronbach α: Study 1: α = 0.88; Study 2: α = 0.89), with responses made on 7-point Likert-type scales (1: strongly disagree, 7: strongly agree).

#### Facebook-Specific Questions

The following questions were administered “Are you a registered user of the social network “Facebook”? [Yes, No]”; “If you are a user of Facebook, how many friends do you currently have on your Facebook profile? If you do not know the exact number, please estimate the number as best as you can.”; “How many hours per week do you use Facebook on average for private purposes?”

#### Additional Facebook-Specific Questions (Project #4 Only)

The following questions were asked: (1) How many people would you label as close friends in your “real” life [i.e., only those friends you meet in person]?, (2) For how long have you had a Facebook account (in months)?, (3) How often do you login to Facebook on average (1: never, 2: every couple of months, 3: several times per month, 4: several times per week, 5: once a day, 6: several times per day), (4) On average, how many minutes do you spend on Facebook per session (i.e., how many minutes does an average Facebook session last)?

#### Rosenberg Self-Esteem Scale (RSES; Project #4 Only)

The RSES (Rosenberg, 1965; German version von Collani and Herzberg, 2003) is a 10-item measure of global self-esteem. It uses a 4-point Likert-type response scale (1: totally disagree, 4: totally agree; α = 0.89).

#### Three-Item Loneliness Scale (TILS; Project #4 Only)

The TILS (Hughes et al., 2003) is a 3-item measure of loneliness that uses a 3-point response scale (1: never/seldom, 2: sometimes, 3: often). The TILS asks about the frequency of certain situations (e.g., “I feel left out.”; α = 0.76).

### Statistical Analyses

Because the variables of number of Facebook friends and time spent on Facebook on average per week were highly skewed (skewness = 1.9 to 16.2) with extreme values, both variables were log-transformed (1 + log_10_) before further analyses (Stieger and Lewetz, 2016; but see Feng et al., 2014). After transformation, skewness was <|1.2|, which can be regarded as acceptable (±2) (Trochim and Donnelly, 2006).

Multicollinearity (i.e., intercorrelation between predictors) is a problem when calculating linear regressions because it can lead to biased estimates. In the present study, multicollinearity was prevalent because the Facebook-specific questions showed substantial correlations (e.g., greater time spent on Facebook was associated with longer average Facebook session, *r* = 0.47). To overcome this problem, a dominance analysis (DA) was conducted (Budescu, 1993; Azen and Budescu, 2003). Through DA, the relative importance of each predictor can be assessed compared to the other predictors in the model. This is done by calculating regression models for all possible combinations of predictors by decomposing the total *R*^2^ (explained variance) into partial, direct, and total effect parts. Partial effects are calculated from all possible combinations of predictors by excluding either one or more predictors from the model. The direct effect can be obtained when the model features only a single predictor (i.e., zero-order correlation with the outcome measure), whereas the total effect is the classical multiple linear regression with all predictors included in the model at once. The results of the DA are *R*^2^ values for each predictor, which are adjusted for shared variances with other predictors (i.e., representing the real explained variance). In the present study, DAs were calculated using the *R* package “yhat” (Nimon and Oswald, 2013). Furthermore, Bayesian Factors (BF) were calculated using the JASP software (Clyde et al., 2011) and the *R* package “BayesFactor” (Morey et al., 2016). BF is akin to betting odds in favor of the alternative hypothesis given the data, i.e., a *BF*_10_ of 15 means that H_1_ is 15 times more probable than H_0_. With respect to the classification scheme of Lee and Wagenmakers (2013), this corresponds to strong evidence for H_1_ (for easier interpretation of *BF*-values smaller than one, *BF*_01_ is sometimes presented).

### Ethics Statement

Participants were from German-speaking countries with clear IRB procedures. The studies were conducted in accordance with the Declaration of Helsinki and the Ethical Guidelines of the University of Vienna. Formal ethics approvals for this type of research (i.e., non-invasive, not affecting the physical or psychological integrity, the right for privacy or other personal rights of interest) are not required according to these guidelines. All participants consented to the terms of the study, which were outlined in detail, preceding the actual questionnaire. As such, providing informed consent was a prerequisite to proceed to the main part of the survey. Participants were explicitly told that they could revoke their consent and withdraw from the study at any time without any personal disadvantages arising from it. Furthermore, anonymity was ensured and no harmful procedures were applied.

## Results

### RQ 1: Are Facebook Users More Satisfied With Their Lives Than Non-Facebook Users?

An independent *t*-test was calculated. As reported in Table 2, neither in Study 1 nor in Study 2 were Facebook users more satisfied with their lives compared to non-Facebook users. In fact, descriptively, the effect was in the opposite direction. Because there were no significant sex differences in life satisfaction or significant correlations between participant age and life satisfaction in both samples, controlling for participants’ sex and age in a two-factorial ANCOVA further corroborated the conclusion that Facebook users were not significantly more satisfied with their lives than non-Facebook users (detailed results omitted for brevity). There was a significant age difference between Facebook users and non-users, but there were no sex-specific effects except for a significant difference in the Study 2 sample, although the BFs were fairly small, i.e., only very weak evidence for H_1_ (Lee and Wagenmakers, 2013).

**TABLE 2:** Differences in life satisfaction and demographics between FB users and non-FB users.

		**FB users**	**Non-FB users**				
		***M* (*SD*)**	***M* (*SD*)**	***t* (*df*)**	***d* (*CI*)**	***BF*_10_**	***Bayes classification***
Study 1	SWLS	5.0 (1.23)	5.1 (1.13)	1.69 (2119)	−0.07 (−0.16, 0.01)	0.20	Moderate H_0_
	Age	30.4 (11.8)	51.0 (14.9)	34.87 (2119)^∗∗∗^	−1.52 (−1.42, −1.61)	>100	Extreme H_1_
Study 2	SWLS	5.2 (1.22)	5.3 (1.10)	1.29 (1230)	−0.08 (−0.20, 0.04)	0.16	Moderate H_0_
	Age	28.7 (9.3)	47.3 (15.6)	26.12 (1230)^∗∗∗^	−1.61 (−1.47, −1.74)	>100	Extreme H_1_

		**% women**	**% women**	**χ^2^ (*df* = 1)**	***OR (CI)***		

Study 1	Sex	56.8	56.0	0.16	1.04 (0.87, 1.23)	0.09	Strong H_0_
Study 2	Sex	61.1	54.0	5.47^∗^	1.34 (1.05, 1.71)	1.49	Anecdotal H_1_

*FB, Facebook; *d*, Cohen *d*; *OR*, Odds Ratio; *CI*, 95% confidence interval. *BF*, Bayes Factor; *BF* for sex is for joint multinomial, ^∗^*p* < 0.05, ^∗∗∗^*p* < 0.001.*

### RQ 2: Are Facebook Users With More Facebook Friends More Satisfied With Their Lives?

Neither in Study 1 nor in Study 2 were significant correlations found (*r* = 0.004, *p* = 0.90, *BF*_01_ = 24.72, strong evidence; *r* = 0.006, *p* = 0.85, *BF*_01_ = 22.69, strong evidence; respectively). Furthermore, there were no significant sex differences for the number of Facebook friends (*t* < 1.68, Study 1: *BF*_01_ = 12.03, Study 2: *BF*_01_ = 3.14, moderate-to-strong evidence), though there was a significant correlation with participant age (Study 1: *r* = −0.514, *p* < 0.001, *BF*_10_ > 100, extreme evidence; Study 2: *r* = −0.449, *p* < 0.001, *BF*_10_ > 100, extreme evidence). Therefore, the initial analyses were re-run, statistically controlling for sex and age differences. Although the correlation approached nominal significance in Study 1 (*r* = 0.060, *p* = 0.06), the correlation was of a tiny effect size (small: 0.1, medium: 0.3, large: 0.5; Cohen, 1988) and the effect clearly diminished in Study 2 (*r* = 0.016, *p* = 0.65). To summarize, having a greater number of Facebook friends was not significantly associated with a higher life satisfaction.

### RQ 3: Are Frequent Facebook Users More Satisfied With Their Lives Than Infrequent Facebook Users?

Interestingly, the time users spent on Facebook was negatively associated with life satisfaction (Study 1: *r* = −0.133, *p* < 0.001, *BF*_10_ > 100, extreme evidence; Study 2: *r* = −0.179, *p* < 0.001, *BF*_10_ > 100, extreme evidence). This weak effect remained – although slightly attenuated in Study 1 – when controlling for possible sex and age differences (Study 1: *r* = −0.109, *p* = 0.001; Study 2: *r* = −0.175, *p* < 0.001).

**FIGURE 1:**
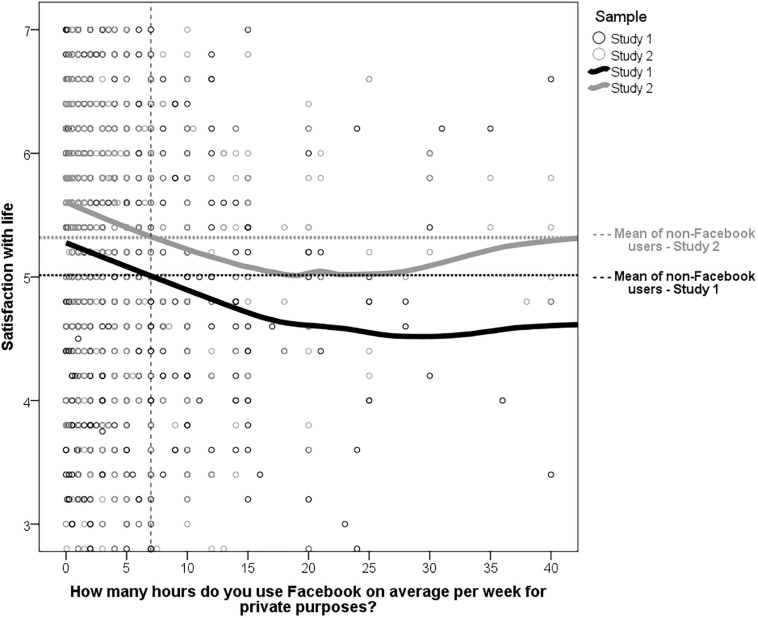
Association between life satisfaction and the hours per week (on average) being on Facebook. For clarity, the non-log-transformed variables were used. The regression lines represent LOESS curves (i.e., local regressions using Epanechnikov kernel, nearest neighbor function = 99%). Dashed horizontal lines represent the mean life satisfaction of non-Facebook users. Dashed vertical line represents the mean hours per week on Facebook where life satisfaction is lower than in the control group (i.e., non-Facebook users).

As can be seen from Figure 1, life satisfaction declined the longer participants were on Facebook during an average week (or *vice versa*). This decline reached floor level around 15 to 20 h per week (for clarity’s sake, the *x*-scale was cropped to 40; the regression lines basically remain parallel to the *x*-scale until the maximum value and applied to both study samples). This was consistent across Studies 1 and 2. Because of the correlational nature of the design, it can only be concluded that there was a significant negative association between the intensity of Facebook usage and life satisfaction, but not that there is a causal relationship.

Although the design is correlational, it can be also regarded as a quasi-experimental design where Facebook ownership as the grouping variable was not randomly assigned. Although this design is less powerful than a real experiment, it offers some clues about possible causal pathways, especially when experiments are difficult to achieve. Therefore, the observed decline in life satisfaction was juxtaposed to the mean of the control group (i.e., participants not having a Facebook account). As can be seen in Figure 1, participants who used Facebook less than approximately 7 h on average per week were indeed more satisfied than participants without a Facebook account (see vertical dashed line in Figure 1), although a decline is also apparent. The same pattern was also found in Study 2.

### Additional Analyses: How Reliable Are the Employed Measures?

One could argue that the items used did not produce reliable results. Indeed, answering the second and third questions about Facebook usage (number of friends, mean duration on Facebook) may have induced a high cognitive burden on the part of participants because they had to think carefully about their answers (especially the third item). Therefore, a retest-design was used (retest after 1 week) in project #2 (*N* = 80) to address this concern empirically. Participants had similar life satisfaction after 1 week (*r* = 0.890; ICC = 0.939), as well as a similar number of Facebook friends (median of difference = 0; *r* = 0.932) and similar mean hours of Facebook usage (median of difference = 0; *r* = 0.878). One participant who stated having no Facebook account had an account a week later and no one left Facebook during this week.

### Additional Analyses: What Is the Best Predictor of Life Satisfaction, Self-Esteem, and Loneliness (Offline Friends, Other Facebook-Specific Usage Indicators)?

Although no significant correlation was found between the number of Facebook friends and life satisfaction, a link might appear when assessing offline friends and administering other Facebook specific questions (e.g., time since creation of Facebook account in months, duration of an average Facebook session, and frequency of logins to Facebook). Furthermore, the question arises as to whether a significant association might appear when measuring other relevant psychological concepts regarding Facebook use, such as self-esteem and loneliness (Mehdizadeh, 2010; Gonzales and Hancock, 2011; Ryan and Xenos, 2011). These additional questions were added to the final project (#4) of Study 2.

As can be seen in Table 3, the number of Facebook friends was not significantly associated with higher life satisfaction, self-esteem, or lower levels of loneliness (all explained variances <0.34%). Interestingly, the number of close *offline* friends was significantly associated with higher life satisfaction, higher self-esteem, and reduced loneliness (explained variances 0.55 to 1.63%). The hours on Facebook on average per week was negatively associated with life satisfaction (non-significant, but 2nd strongest predictor in the DA with 2.00% explained variance) and self-esteem (significant and again 2nd strongest predictor with 1.80% explained variance). The possession of a Facebook account showed no substantial effects (all explained variances <0.27%). The duration of an average Facebook session had a negative – albeit non-significant – association with life satisfaction, which explained 1.08%, representing the 4th strongest predictor of life satisfaction. Furthermore, the frequency of how often participants logged-in to Facebook was the strongest predictor for all three psychological constructs (explained variances 1.40 to 2.88%).

**TABLE 3:** Results of the linear regression, dominance, and Bayes Factor analyses (project #4).

	**Life satisfaction**			**Self-esteem**			**Loneliness**		
	**β**	***BF*_10_**	**Dominance *R*^2^ [%]**	**β**	***BF*_10_**	**Dominance *R*^2^ [%]**	**β**	***BF*_10_**	**Dominance *R*^2^ [%]**
Age	–0.01	0.10	0.03	0.11^∗^	22.27	1.37	−0.10^∗^	1.52	0.95
Sex	0.03	0.12	0.09	–0.04	0.13	0.14	0.10^∗^	1.39	0.99
Number of Facebook friends.^1)^	0.07	0.10	0.34	0.05	0.16	0.15	–0.07	0.10	0.22
Number of close offline friends.^1)^	0.13^∗∗^	4.49	1.63	0.08‡	0.20	0.55	–0.11^∗∗^	2.49	1.29
Hours on Facebook on average per week.^1)^	–0.10	>100	2.00	−0.14^∗^	>100	1.80	–0.04	0.12	0.10
Possession of Facebook account in months.^1)^	0.05	0.18	0.27	0.05	0.11	0.18	0.01	0.10	0.02
How long takes a session on average in minutes?^1)^	–0.08	10.79	1.08	0.02	0.52	0.31	0.03	0.10	0.06
How often do you log in onto Facebook?	–0.18^∗∗^	>100	2.88	−0.11^∗^	>100	2.11	0.15^∗∗^	2.73	1.40
	*F*(8,569) = 5.99, *p* < 0.001; *R*^2^ = 7.8%			*F*(8,569) = 4.76, *p* < 0.001; *R*^2^ = 6.3%			*F*(8,569) = 3.63, *p* < 0.001; *R*^2^ = 4.8%		

*Coding of Sex: 1, male; 2, female. ‡*p* < 0.10, ^∗^*p* < 0.05, ^∗∗^*p* < 0.01 (two-tailed). ^1)^Variables were log-transformed (1 + log_10_). Bayes classification for *BF*_10_: >100, extreme evidence for H_1_; 30–100, very strong evidence for H_1_; 10–30, strong evidence for H_1_; 3–10, moderate evidence for H_1_; 1–3, anecdotal evidence for H_1_; 1, no evidence; 1-0.33, anecdotal evidence for H_0_; 0.33-0.10, moderate evidence for H_0_; 0.10-0.03, strong evidence for H_0_; 0.03-0.01, very strong evidence for H_0_; <0.01, extreme evidence for H_0_.*

## Discussion

In general, Facebook users were not significantly more satisfied with their lives than non-Facebook users and having more Facebook friends did not seem to go hand-in-hand with higher life satisfaction or *vice versa*. However, using Facebook more often appeared to be associated with significantly lower satisfaction with life, albeit with a small effect size. These conclusions were stable across both samples (Study 1 and Study 2). These results are interesting in at least two respects.

First, social contact should normally facilitate our satisfaction with life. Humans are social beings: we have a drive to build social groups and to communicate. Effects of sensory deprivation and social isolation are well known and can lead to detrimental psychological effects (e.g., depression; Cacioppo et al., 2010). Nevertheless, in the present studies, no positive effect of having a Facebook account or having more Facebook friends was found. Interestingly, in an additional regression analysis of a large subsample (Study 2; project #4; *N* = 578; Table 3), the number of close *offline* friends was positively associated with life satisfaction and self-esteem (although only at a 10% significance level), and negatively with loneliness, but the number of Facebook friends still failed to show any significant effects. This is interesting, because online and offline friends often overlap. Although firm conclusions cannot be drawn from this cross-sectional design and the small effect sizes, it could be that offline friends are slightly more relevant in terms of life satisfaction than Facebook friends.

Second, social interaction *per se* should not only facilitate our satisfaction with life, but should also be influenced by the intensity of said social interaction. In the present case, a negative association was found, i.e., the more time spent on Facebook, the lower the satisfaction with life and *vice versa* (for an early description of a similar paradox; Kraut et al., 1998). Again, in a supplementary analysis, other operationalizations of the intensity of Facebook usage were employed (Study 2; project #4). Neither the general period of Facebook use (in months), nor the duration of an average Facebook session, showed any significant positive associations. Interestingly, the number of logins to Facebook again showed negative effects, i.e., lower satisfaction with life and self-esteem, and higher loneliness. This negative effect of Facebook login frequency was highest in terms of explained variances compared to all the other predictors in the model (see Table 3) for all three constructs under investigation (satisfaction with life, self-esteem, loneliness), though again effect sizes were small.

One possible explanation for this pattern of results comes from addiction research. Although still controversially discussed, it seems that the Internet in general has addictive qualities (Young, 1998; Pawlikowski et al., 2014). One of the best predictors of most problematic Internet uses (e.g., Internet, Gaming, SNS) is the average duration of usage for private purposes (Stieger and Burger, 2010). Furthermore, greater online activity and more logins to Facebook were associated with negative life outcomes (e.g., self-esteem; Mehdizadeh, 2010). Although problematic Internet use was not measured in the present studies, the negative association of Facebook usage with life satisfaction might indicate addictive tendencies of participants. To sum up, although Facebook comes with the advantage of higher social connectedness, it does not seem that this makes us more satisfied with our lives (Kross et al., 2016). On the contrary, it appears to possess features that either spur problematic Internet use or attract people with addictive tendencies (Ryan and Xenos, 2011).

### Limitations

The present studies are limited by the fact that Facebook usage was globally defined without any differentiation. However, today it is known that there might be differences in motivation and behavior between users who are active vs. passive on Facebook (Verduyn et al., 2015; Appel et al., 2016). Nevertheless, the main research question was how the overall Facebook use is associated with users’ life satisfaction. Another limitation stems from the correlational design, i.e., conclusions about causality cannot be drawn. This limitation was one of the reasons for also recruiting non-Facebook users in order to have a control group. Yet, the design was quasi-experimental in nature. Nevertheless, research using experience sampling designs (i.e., longitudinal diary studies) have found a link between Facebook usage and lowered subjective well-being (Kross et al., 2016). Furthermore, an experiment with Facebook quitters found that taking a break from Facebook led to increased life satisfaction and more positive emotions compared to non-quitters (Tromholt, 2016). Furthermore, the focus of the present studies was on Facebook usage *intensity*, rather than the *quality* of interactions. Because it is well known that positive interactions best meet belonging needs (Baumeister and Leary, 1995), future research should analyze this in more detail. Another point is the validity of the Facebook usage measure. Past research suggests that this self-reported SNS use item is not particularly accurate (Ellis et al., 2019; Foster and Jackson, 2019; Orben and Przybylski, 2019; Orben et al., 2019a, b). Although I found high test-retest reliability for scores on this measure in sample #4 of Study 2, it may still be the case that the item is affected by, for example, recall biases.

### Future Directions

Future research might assess the motivations to use Facebook. It is conceivable that passive Facebook users (e.g., shy or lonely people, people with limited offline social contacts) are more prone to the negative effects of Facebook compared to active Facebook users who see Facebook as an extension of, and not surrogate for, their real-life offline social networks (Verduyn et al., 2015; Appel et al., 2016). Future research might also differentiate what is meant by “friends” in a more fine-grained manner. With respect to offline friends, we usually have a clearer definition of whom we call friend or not, but online any acquaintance is defined as friend through the terminology of the respective social media platforms. Furthermore, most offline friends are probably also online friends on Facebook, provided that these persons have a Facebook account. Asking about real Facebook friends might result in different conclusions compared to asking about Facebook friends in general (Grieve et al., 2013). Although real Facebook friends still need to be defined (e.g., someone with whom there are regular Facebook interactions), further research might benefit from this avenue for future research.

## Data Availability Statement

The datasets generated for this study are available on request to the corresponding author.

## Ethics Statement

Ethical review and approval was not required for the study on human participants in accordance with the local legislation and institutional requirements. The patients/participants provided their written informed consent to participate in this study.

## Author Contributions

SS was the principal investigator, conceived the study, contributed to the study design, data analyses, data management, and writing of the manuscript.

## Conflict of Interest

The author declares that the research was conducted in the absence of any commercial or financial relationships that could be construed as a potential conflict of interest.
