# Supplementary material for: Facebook Usage and Life Satisfaction
Source: Front Psychol. 2019 Nov 29;10:2711. doi: 10.3389/fpsyg.2019.02711 (PMC6896249; doi:10.3389/fpsyg.2019.02711)
Supplement: Supplementary file 1 [file Table_1.DOCX]

Supplementary Materials

# Power analysis

A power analysis was calculated. Due to the inconclusive results from previous research about the effect size of a possible relationship between Facebook usage and life satisfaction, the power analysis was based on a small effect size (*r* = 0.10; 80% power, α = 5%, two-sided). The calculated minimum sample size requirement was *N* = 782.

# Non-independence effects

To test for possible non-independence effects [1,2] because of the convenience sampling approach (i.e., different research assistants may have recruited samples with varying satisfaction with life), an intraclass correlation coefficient (ICC) was calculated (in Study 1 only). The formula from Kenny et al. [1] and the approach of Grawitch and Muntz [2] were used, which resulted in an ICC of .016. This means that most of the variance in life satisfaction was within the recruited research assistant groups rather than between groups, i.e., recruited sub-samples did not substantially differ across research assistants.

# Measurement invariance

The degree to which the measurement of life satisfaction using the Satisfaction with Life Scale was equivalent across the groups of Facebook users and non-users was assessed (i.e., measurement invariance).

Measurement invariance is tested by comparing models with different constraints across groups. In a baseline model, factor loadings and intercepts can vary freely across groups (configural invariance). In the next model, factor loadings are fixed across groups and again model fit indices are calculated. If the model fit indices do not substantially differ, metric invariance (also called weak invariance) is established. If model fit indices worsen through constraining factor loadings, it suggests that the factor loadings are not similar across both groups. A further model additionally constrains the intercepts across groups (scalar or strong invariance). Again, if the new model does not worsen fit indices compared to the previous model, we can assume that intercepts are equal across groups. In a final model, the residuals are also fixed. This is the most stringent model (strict invariance).

In order to decide if the more stringent model worsens model fit, differences in Root Mean Square Error of Approximation (RMSEA) and Comparative Fit Index (CFI) are analyzed. Chen [3] recommended a ΔCFI < .01 and a ΔRMSEA < .015 in order to fulfil the criterion of measurement non-invariance. In general, according to Hu and Bentler [4], RMSEA should be lower than .08 and CFI higher than .95 in order to have an acceptable model fit.

Table S1. Goodness-of-fit statistics for tests of measurement invariance with the grouping variable Facebook user vs. non-user

|  | CFI | ΔCFI | RMSEA | ΔRMSEA |
| --- | --- | --- | --- | --- |
| Configural invariance | .990  .980 |  | .070  .106 |  |
| Metric invariance (+ loadings fixed) | .986  .981 | -.004  .001 | .072  .087 | .001  -.019 |
| Scalar invariance (+ Intercepts fixed) | .981  .981 | -.005  -.001 | .073  .078 | .001  -.009 |
| Strict invariance (+ Residuals fixed) | .977  .980 | -.004  -.001 | .071  .071 | -.002  -.007 |

*Note*. First-line entries = Study 1, second-line entries = Study 2.

As can be seen in Table S1, in general more stringent models did not lead to substantially worse model fits across groups in both studies (Study 1 and 2). Interestingly, the basic model in Study 2 had poor fit to the data (RMSEA = .106) but was substantially improved (ΔRMSEA = -.019) by restraining the loadings across groups. This means that the factor structure for Facebook users was somewhat different from that of non-users. A closer look at model fit indices for Facebook users vs. non-users revealed that RMSEA for non-Facebook-users was less-than-adequate with a value of .148, although the CFI was acceptable (.959). Comparing standardized factors loadings across both groups did not reveal any substantive problems with individual items.

# References

1. Kenny DA, Mannetti L, Pierro A, Livi S, Kashy DA. The statistical analysis of data from small groups. Journal of Personality and Social Psychology 2002; 83:126-37.
2. Grawitch MJ, Munz DC. Are your data nonindependent? A practical guide to evaluating nonindependence and within-group agreement. Understanding Statistics 2004; 3(4):231-57.
3. Chen FF. Sensitivity of goodness of fit indexes to lack of measurement invariance. Structural Equation Modeling: A Multidisciplinary Journal 2007; 14:464-504.
4. Hu LT, Bentler PM. Cutoff criteria for fit indexes in covariance structure analysis: Conventional criteria versus new alternatives. Structural Equation Modeling: A Multidisciplinary Journal 1999; 6:1-55.
